# Quantify retinal structure in high-altitude residents with and without high altitude polycythemia

**DOI:** 10.1186/s12886-022-02674-7

**Published:** 2023-01-03

**Authors:** Jinlan Ma, Haoyu Niu, Changjing Han, Yi Qu

**Affiliations:** 1 Department of Ophthalmology, Affiliated Hospital of Qinghai University, Xining, China; 2 Department of Health Care, Qilu Hospital of Shandong University, No. 107, Wenhuaxi Road, Jinan, 250012 China

**Keywords:** High altitude polycythemia, High-altitude retinopathy, Hypoxia, Optical coherence tomography, Central retinal vein equivalent

## Abstract

**Background:**

To assess retinal structural parameters in high-altitude (HA) residents with and without high altitude polycythemia (HAPC) and to elucidate the relationship between retinal structural parameters and hemoglobin (HGB).

**Methods:**

This cross-sectional study included 55 HAPC patients and 52 healthy HA residents. Retinal structural parameters included retinal nerve fiber layer (RNFL) thickness, optic nerve head (ONH) parameters and retinal vessel diameter. RNFL thickness were acquired from spectral domain optical coherence tomography (SD-OCT) built-in software. ONH parameters including neuroretina rim height, cup area, disc area and vertical cup/disc ratio were obtained by OCT built-in software and ImageJ software. Retinal vessel measurements including central retinal artery equivalent (CRAE), central retinal vein equivalent (CRVE) and AVR (artery/vein ratio) were calculated by revised formulas for summarizing retinal vessel diameters. All parameters were compared between HAPC group versus healthy HA group. The associations between retinal parameters and HGB were assessed by Pearson correlation analyses.

**Results:**

In comparison of HAPC group versus healthy HA group, RNFL thickness was thicker in the nasal quadrant of the optic disc in HAPC group (74.82 ± 14.4 VS. 66.06 ± 13.71 μm, *P* = 0.002). Bigger disc area and bigger cup area were also observed in HAPC group (all *P* < 0.05). Meanwhile, the value of CRVE was higher in HAPC group which suggested that retinal veins dilated significantly in HAPC patients (*P* < 0.001), however, CRAE and AVR were comparable between groups. Pearson analyses revealed that HGB was positive correlated with CRVE in HAPC group (*r* = 0.469, *P* = 0.003).

**Conclusions:**

long-term HA exposure secondary HAPC could result in thickened RNFL, enlarged ONH and dilated retinal veins. Moreover, increased blood viscosity caused by HGB should be responsible for dilated veins, but not for thickened RNFL and enlarged ONH. This study deepens the understanding of the impact of HA environment on retina.

## Backgrounds

Hypoxia and hypobaric nature in high-altitude (HA) area could cause pathologic changes in cardiovascular system, respiratory system and nervous system [1]. The retina is one of the most active tissues, hence pathological level of hypoxia may firstly cause functional and structural changes in retina [2–8]. Hemorrhages, cotton wool spots, and papilledema that occurred during acute exposure to HA environment are known as high-altitude retinopathy (HAR) [9–11]. Structural changes in HAR are attributed to possible ocular vascular dysregulation, however, the etiopathogenesis underlying HAR is not defined [12, 13].

Structural changes in HAR usually appeared in RNFL thickness, optic disc and retinal vessels. Tian et al. observed significantly increasing in retinal nerve fiber layer (RNFL) thickness after rapid ascent to HA environment [14]. Clarke et al. showed RNFL thickened more obviously than the outer layers of retina during acute exposure to HA [15]. And Fischer et al. indicated that acute exposure to HA does not result in macular edema, but slight increase in perimacular RNFL thickness [16]. RNFL is a hot topic of retinal study as the sources of optic nerve, and RNFL thickness was reported to be related with acute mountain sickness(AMS) [15, 17].

Optic disc edema (ODE) was detected commonly in AMS [18–20], however, the relationship between ODE and AMS was controversial [21]. And the pathophysiological mechanism of ODE remains unclear [22].Examination of the optic nerve head (ONH) could be a perfect method to non-invasively and directly check the state of brain and the nervous system.

There was evidence that during acute HA exposure, vascular resistance decreased and vessels dilated to satisfy the increasing oxygen demand, which could result in vasogenic cerebral edema and capillary overperfusion [23]. Clarke et al. [15] and Merz et al. [23] both discovered that retinal veins dilated significantly in healthy people during acute exposure to HA environment. The hypothesis about increased blood flow also focused on the diameter of vessel. Standardized protocols [24, 25] and revised formulas which developed by Knudtson et al. [26]for measurement of central retinal artery equivalent (CRAE)and central retinal vein equivalents (CRVE) could provide more precise and consistent estimates of retinal vessel diameter in eye.

All of the studies mentioned above reported HAR in condition of acute hypoxia during rapid ascent to HA environment. Actually, there are a large number of people live with chronic hypoxia in HA environment throughout the world, moreover, high altitude polycythemia (HAPC) is one of the most common chronic diseases in HA residents and increased blood viscosity caused by HAPC has been suggested as a risk factor of HAR [27], hence, studying the retinal structural changes in these people may provide a new insight into the effect of long-term HA exposure on the retina.

Our study aimed to compared RNFL thickness, ONH parameters and retinal vessel diameters between HAPC patients and healthy HA residents using optical coherence tomography (OCT) in-built software, ImageJ software (National Institutes of Health [NIH], Bethesda, Maryland, USA) and revised formulas of retinal vessels in order to assess the effect of long-term HA exposure on retina. In addition, the relationship between these metrics and HGB were detected, as a proxy for etiopathogenesis of high-altitude related retinopathy.

## Methods

### Participants

This cross-sectional study was performed from January 2020 to December 2021 in Affiliated Hospital of Qinghai University, which is located in Qinghai province, lies in the Qinghai-Tibet Plateau. This study followed the guidelines of the Declaration of Helsinki and was approved by the Ethics Committee of Affiliated Hospital of Qinghai University. Written consents were obtained from all participants after being given a detailed explanation of the study.

55 HAPC patients and 52 age-matched healthy HA residents formed the basis for the current analysis. We traveled to two counties of Qinghai Province to collect HAPC patients and healthy HA subjects, one was Maqing County (average altitude 3730 m), another was Dawu County (average altitude 3719 m). And all data were measured in the local hospital. All of the participants were local residents who have been living in HA areas for more than ten years and with no visit to low altitude region in at least 1 year prior to this study. Hemoglobin (HGB) concentration was assessed by automated hematology analyzer (Sysmex XE 2100, Kobe, Japan). The inclusion criteria for HAPC subjects as follows: HGB concentration ≥ 190 g/L for female or ≥ 210 g/L for male [28, 29]; the history of HAPC more than one year; without any therapy during last three months, without chronic pulmonary diseases or other underlying chronic medical conditions that worsen the hypoxemia, excluded other types of polycythemia and no signs or history of cardiovascular diseases, diabetes mellitus, renal insufficiency or other concomitant disease; age between 18 and 65 years. The inclusion criteria for healthy HA participants as follows: HGB were 120-160 g/L for male and 110-150 g/L for female; without history of any chronic diseases; age between18 and 65 years and with healthy status in eye.

All participants underwent detailed ophthalmic examination by two retinal specialists including slit lamp examination; computer optometry; IOP measurement; fundoscopy and optic disc cube 200 × 200 OCT scan. Exclusion criteria were: cloudy refractive structure; IOP ≥ 21 mmHg; history of ocular disease, trauma, or surgery; spherical equivalent (SE) of exceeding ± 3.0 diopters; abnormal fundus status such as retinal hemorrhage, cotton wool spots and hard exudates.

### Quantitative analysis of RNFL

OCT was performed with Zeiss Cirrus HD-OCT 5000 (Carl Zeiss Meditec, Inc., USA) after pharmacologic mydriasis with 1% tropicamide. Only high-quality images (signal strength ≥ 7) were included. Each participant underwent optic disc cube 200 × 200 scan to measure RNFL thickness. The scan protocol included a peripapillary circular scan with a diameter of 3.4 mm centered on the disc. The built-in software allows the mapping of the thickness data according to both quadrant-by-quadrant and clock-hour analyses. RNFL thickness in the superior, inferior, nasal and temporal quadrants of the optic disc were collected.

### Quantitative analysis of ONH parameters

**Fig. 1:**
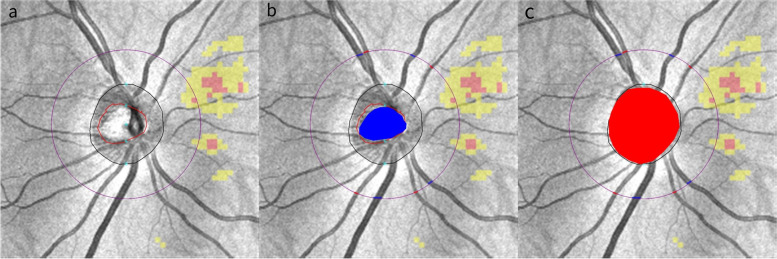
Cup area and disc area in OCT images. a: original grayscale optic nerve head (ONH) images; b: cup area is manually marked as blue color; c: disc area is manually marked as red color. For each peripapillary quadrant, the largest diameter vein and artery which crossed the circle were marked. Veins were marked as blue and arteries were marked as red. Finally, the diameters were put into equation to calculate CRAE/CRVE/AVR

There were obvious errors in cup and disc segmentation in the OCT built-in software, so most of the ONH parameters were obtained by ImageJ software except for neuroretina rim height. The disc area, cup area and vertical cup/disc ratio were semi-automatically calculated by ImageJ: First, set scale, set 6 mm as known distance of width of ONH scan images; Second, cup and disc segmentation, “polygon selection”and “color picker” tools were used to mark cup area and disc area as region of interest (ROI) manually and measured in mm^2^; Third, vertical cup/disc ratio was expressed as ratio of vertical diameter of cup to vertical diameter of disc. Only the right eyes were included. Figure 1a was the original grayscale scan, Fig. 1b and c showed the veritable cup area and disc area marked by ImageJ.

### Retinal vessel diameter measurement

The ONH cube scan at 6 mm × 6 mm field of view was acquired and a circle with a diameter of 3.4 mm was centered on the ONH. The largest veins and arteries in superior nasal quadrant, inferior nasal quadrant, superior temporal quadrant, and inferior temporal quadrant were marked by two independent, trained ophthalmologists. Image brightness and contrast were adjusted suitable to detect the borders of the vessels. “Set scale” and “straight” tools in ImageJ software were used to calculate the diameters of the largest vein and artery passing through the circle in each quadrant [30]. As Parr et al. [24] and Hubbard et al. [25] suggested, blood flow is proportional to the lumen of vessels rather than caliber**,** and the number and pattern of branching of the larger vessels influence the diameter of trunk vessel**,** the Parr-Hubbard method summarized the individual retinal vessel measurements into the central retinal artery equivalent (CRAE) and central retinal vein equivalent (CRVE). Then Knudtson et al. [26] developed revised formulas that summarize the CRAE and CRVE as follow:

$$Arterioles:\omega=0.88\ast(W_1^2+W_2^2\;){1/2}^{}$$

$$Arterioles:\omega=0.95\ast(W_1^2+W_2^2\;){1/2}^{}$$

w_1_ and w_2_ are the widths of the narrower branch and the wider branch respectively, and ${\varvec{\omega}}$ is the parent trunk. Using above formulas, including four largest arteries and the four largest veins, we used an iterative procedure to obtain CRAE/CRVE. For example, we calculated the diameter of largest vein in each quadrant in ONH cube scan:0.148, 0.101, 0.195 and 0.204 mm. First put 0.204 and 0.101 into above equation and yield 0.22 mm. Similarly pair up 0.195 and 0.148 to yield 0.23 mm. Finally, pairing up o.22 and 0.23 yield 0.3 mm as CRVE. AVR (artery/vein ratio) was the ratio of CRAE to CRVE. Figure 1b shows the selected largest veins and arteries in four quadrants.

### Statistical analysis

Data were analyzed using SPSS version 23 software (SPSS; IBM, Armonk, NY, USA).

Continuous measurements were reported with the mean ± standard deviation (SD) and categorical values were shown in the forms of frequency. Independent-samples t-test was used in comparison for continuous variables and Chi-square test for categorical variable. Pearson correlation analyses were performed to explore the association between HGB and ocular parameters in HAPC patients. Only right eyes were included. A *p*-value < 0.05 was considered statistically significant.

## Results

Baseline characteristics are listed in Table 1. 4 participants in HAPC group and 2 participants in control group were excluded because of low-quality OCT images. 51 eyes from 51 HAPC patients and 50 eyes from 50 healthy HA residents were included finally. There were no statistical differences in age or gender between the HAPC group and healthy HA group (*P* > 0.05). BMI was statistically higher in healthy HA group. The HGB were 219.84 ± 9.32 g/L in HAPC group and 129.8 ± 17.52 g/L in healthy HA group (*P* < 0.001). The HA living history was comparable between groups and the history of HAPC was 3.2 ± 1.2 years for observation group.

**Table 1:** Demographics of participants

	**HAPC**	**Healthy HA**	***P*-value**
Number of subjects	51	50	/
Age(years)	49.51 ± 9.06	47.04 ± 10.3	0.203
Gender(male/female)	40/11	35/15	0.11
BMI	22.31 ± 2.45	23.56 ± 2.08	0.007^*^
Ethnicity (Tibetan/Han)	15/36	21/29	/
HA living history (year)	21.4 ± 10	23.2 ± 7.6	0.3
History of HAPC (year)	3.2 ± 1.2	/	/
IOP	12.63 ± 2.48	11.85 ± 1.83	0.07
HGB(g/L)	219.84 ± 9.32	129.8 ± 17.52	< 0.001^*^

*HAPC* High altitude polycythemia, *HA* High altitude, *HGB* Hemoglobin, *BMI* Body mass index; * statistically significant

### OCT parameters

As shown in Table 2, in the nasal quadrant of the optic disc, RNFL thickness was significant thicker in HAPC group than the healthy HA group (74.82 ± 14.4 VS. 66.06 ± 13.71 μm, *P* = 0.002), however, there were no significant differences in RNFL thickness in the remaining quadrants of the optic disc between two groups(*P* > 0.05). The average RNFL thickness was 102.93 ± 11.59 μm in HAPC group and 100.86 ± 10.1 μm in control group without significant difference between two groups (*P* = 0.34).

**Table 2:** OCT parameters in HAPC group and healthy HA resident group

	**HAPC**	**Healthy HA**	***P*-value**
**RNFL thickness**			/
Eyes(n)	51	50	/
Mean(μm)	102.93 ± 11.59	100.86 ± 10.1	0.341
Superior(μm)	130.33 ± 17.20	128.1 ± 19.12	0.539
Inferior(μm)	133.06 ± 22.63	132.56 ± 13.96	0.894
Nasal(μm)	74.82 ± 14.4	66.06 ± 13.71	0.002^*^
Temporal(μm)	73.51 ± 11.82	76.72 ± 11.17	0.164
**ONH parameters**			/
Eyes(n)	43	42	/
neuroretina rim height(μm)	494.86 ± 157.42	461.42 ± 137.36	0.3
cup area( mm^2^)	0.56 ± 0.27	0.44 ± 0.23	0.03^*^
disc area(mm^2^)	2.39 ± 0.43	2.12 ± 0.38	0.003^*^
vertical cup/disc ratio	0.48 ± 0.15	0.42 ± 0.16	0.07
**Retinal vessel diameters**			
Eyes(n)	47	33	/
CRVE	0.25 ± 0.03	0.2 ± 0.02	< 0.001*
CRAE	0.16 ± 0.12	0.12 ± 0.04	0.07
AVR	0.63 ± 0.43	0.6 ± 0.26	0.72

*HAPC* High altitude polycythemia, *HA* High altitude, *OCT* Optical coherence tomography, *RNFL* Retinal nerve fiber layer, *ONH* Optic nerve head, *CRVE* Central retinal vein equivalent, *CRAE* Central retinal artery equivalent, *AVR* Artery/vein diameter ratio; * statistically significant; *P*-value were obtained from independent-samples t-test

In the ONH analysis section, some eyes were excluded because of difficulties in cup and disc segmentation in these images. Finally, 43 eyes in HAPC group and 42 eyes in control group were included. The outcomes of ONH parameters were displayed in Table 2. The results disclosed that disc area in the HAPC group (2.39 ± 0.43mm^2^) was significant bigger than healthy HA group (2.12 ± 0.38mm^2^, *P* = 0.003) as well as the cup area was significant bigger in HAPC group (0.56 ± 0.27mm^2^ VS. 0.44 ± 0.23mm^2^, *P* = 0.03^)^ The remaining ONH parameters including neuroretina rim height and vertical cup/disc ratio were comparable between HAPC group and control group (all *P* > 0.05).

### Retinal vessel diameters

In this section, 47 eyes in HAPC group and 33 eyes in healthy HA group were included because we can clearly distinguish between veins and arteries in those images. CRVE and CRAE calculated by revised formulas took into account the relation between parent trunk vessels and their branches. In our study, we measured the calibers of the largest vein and artery in each quadrant as branch vessels and calculated CRVE/CRAE by above formulas. The results showed CRVE was higher in HAPC patients (0.25 ± 0.03 VS.0.2 ± 0.02, *P* < 0.001), however, no significant difference in CRAE was found between groups (*P* = 0.07). AVR which known as an indicator of retinal arteriosclerosis was comparable between groups(*P* = 0.7) (Table 2).

### Pearson correlation analyses

**Fig. 2:**
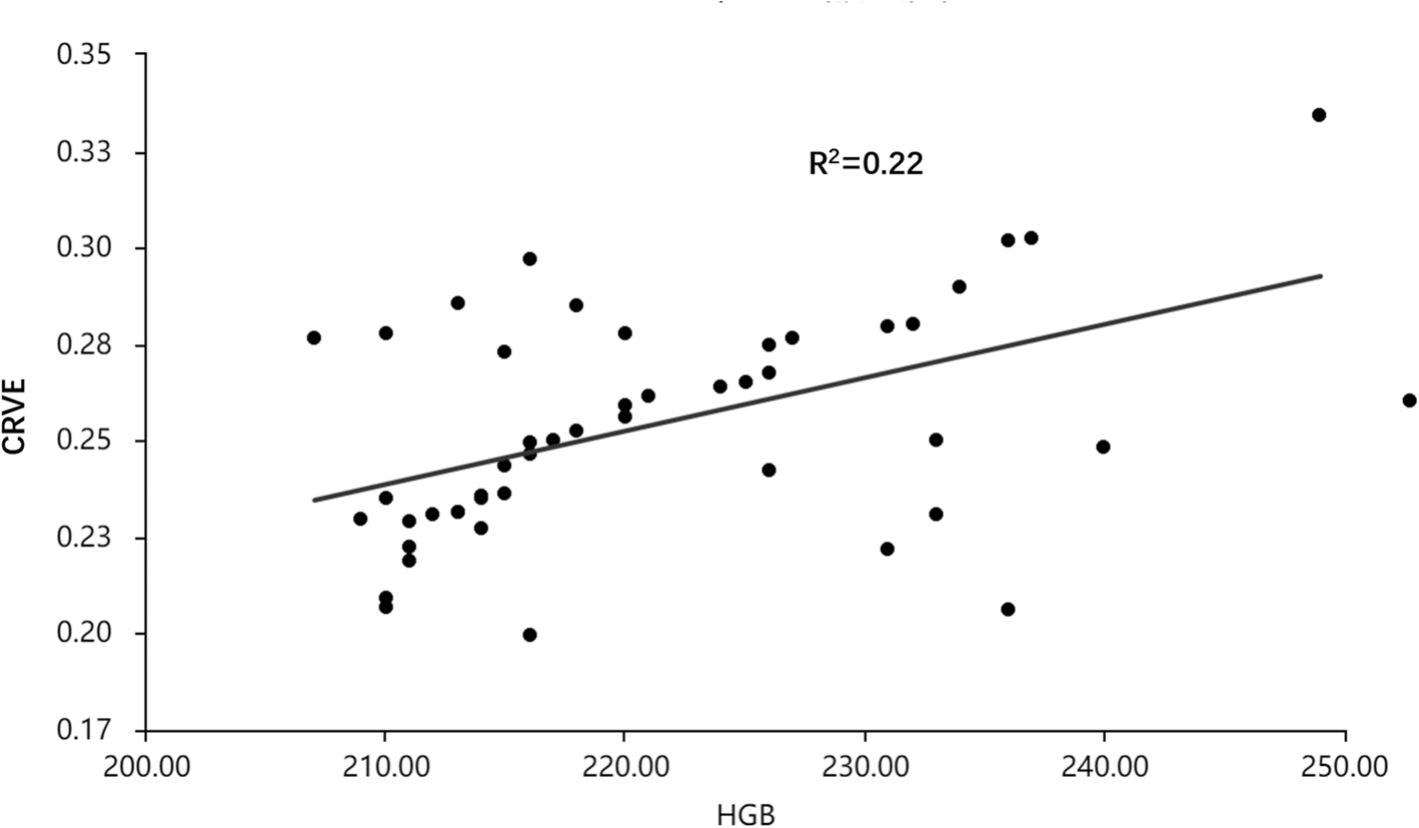
Scatter plot between HGB and CRVE. Central retinal vein equivalent (CRVE) was positive correlation with HGB in HAPC patients (*R*.^2^ = 0.22)

We performed Pearson correlation analyses to explore the association between HGB and OCT parameters in HAPC group. The results suggested that there was significant correlation between CRVE and HGB in HAPC group (*r* = 0.469, *P* = 0.003). Other parameters were independent of HGB (all *P* > 0.05) (Table 3). The scatter plot suggested positive correlation between HGB and CRVE(*R*^2^ = 0.22) (Fig. 2).

**Table 3:** Pearson’s correlations between HGB and OCT parameters in HAPC group

OCT parameters	HGB	
	r	*P*-value
Mean RNFL thickness(μm)	0.226	0.617
Superior RNFL thickness(μm)	0.276	0.052
Inferior RNFL thickness(μm)	0.106	0.462
Nasal RNFL thickness(μm)	0.176	0.221
Temporal RNFL thickness (μm)	0.073	0.617
neuroretina rim height(μm)	0.116	0.46
cup area(mm^2^)	0.133	0.8
disc area(mm^2^)	0.152	0.6
vertical cup/disc ratio	-0.07	0.09
CRVE	0.469	0.003*
CRAE	-0.032	0.8

*HAPC* High altitude polycythemia, *OCT* Optical coherence tomography, *RNFL* Retinal nerve fiber layer, *V* Vein, *A* Artery; * statistically significant

## Discussion

This study disclosed the effect of long-term HA exposure on retinal structure. This paper is the first evidence of thickened RNFL in nasal quadrant, enlarged optic disc and dilated retinal veins in HAPC patients. We first adopted revised formulas in ONH images of OCT scan to calculate CRVE and CRAE and to estimate vessel caliber in HAPC patients.

The results of our study demonstrated that RNFL thickness in the nasal quadrant of the optic disc was significant thicker in HAPC group, which was consistent with prior studies: Clarke et al. reported RNFL thickness increased significantly for all four quadrants in 20 healthy climbers during acute ascent to Margherita Hut [15]. Another study reported significantly thickened RNFL in the nasal and temporal quadrants of the optic disc, whilst obvious thinned RNFL in the inferior quadrant of optic disc after exposure to the HA environment of Tibet [14]. Hence, hypoxia in HA area may be a factor of changed RNFL thickness and the changes of RNFL in different quadrants maybe different. The hypothesis regarding the pathophysiology of thickened RNFL in HAPC patients was supposed that increased retinal blood flow and decreasd arterial oxygen partial pressure (PaO_2_) result in thickened RNFL. The retina has two blood circulation systems: the choroidal circulation system which mainly supplies the outer retina is insensitive to hypoxia and not much regulated by PaO_2_ [23]; whereas the retinal circulation which mainly supplies the inner retina (RNFL and ganglion cell layer) is closely correlated with tissue oxygen and a decline of PaO_2_ could cause an increasing in retinal blood flow immediately [31]. In HAPC patients, the ability of altitude adaptation is overload, they may undergo mild to moderate hypoxia and lower PaO2 which could result in increased blood flow and thickened RNFL. Moreover, RNFL is particular sensitive to hypoxia because of the greatest vascular density in this layer: 1. most of the large vessels pass through this layer and 2, the radial peripapillary capillary plexus additionally support this layer, hence, vascular expansion could cause thickening of RNFL especially [32, 33]. In our study, it was interesting that the thickened RNFL was found in nasal quadrant. Usually, the RNFL was thickest in the superior and inferior quadrants and thinner in the temporal and nasal quadrants. However, RNFL thickened most in temporal and nasal quadrants no matter during acute HA exposure [14] or chronic HA exposure. This phenomenon could be explained by: the radial peripapillary capillary plexus in the nasal of disc result in higher vessel density and more glial cells in the nasal quadrant, hence, make it very sensitive to hypoxia. We need more pathophysiological study to verify our hypothesis in the future.

Our study showed bigger cup area and bigger disc area in HAPC patients which could be diagnosed as subclinical optic disc edema(ODE) as Ascaso et al. suggested [17]. In fact, ODE was related to various diseases such as infection, toxicosis, ischemia and so on [21], whereas papilledema was related to raised intracranial pressure (ICP) especially. For a long time, researchers suggested HA related ODE should be referred as papilledema because it caused by higher ICP: hypoxia environment in HA could disrupt blood–brain-barrier and initiate extracellular vasogenic edema, then vessels began to leak and ICP raised, which has been considered to play a pivotal role in the development of papilledema and AMS [34]. However, following studies investigating the relationship between AMS and increased ICP did not support this hypothesis [35, 36]. Wilson et al. first proposed that AMS would be attribute to restricted cerebral venous outflow which could result in distension of large cerebral veins and increased inflow [37, 38]. And Fischer et al. speculated that HA related ODE was not a result of increased ICP because that this level of hypoxia in AMS was not sufficient to induce such high ICP, but dilated retinal vessels induced increased blood flow which had been considered as a mark of HAR could be related to ODE [39]. A study on cerebral anoxia by MRI certified that hypoxia disturbed axonal transport and induced cytotoxic intracellular edema, which may contribute to ODE [40]. In our study, the obvious dilated veins are evidence of disturbed venous outflow and increased retinal blood flow, which could lead to capillary overperfusion and ODE. Although we could not confirm whether there is higher ICP in HAPC patients, we insisted that chronic hypoxia in HAPC patients is insufficient to cause higher ICP, and the subclinical ODE are caused by disturbed venous outflow. Moreover, we do not know the clinical meaning of subclinical ODE in HAPC patients, which need more research in the future.

We adopted revised formulas developed by Knudtson et al. to summarize the retinal vessel diameters [26]. The revised formulas correlate highly with the previously used Parr-Hubbard formulas, but offer the advantages of being more robust against variability in the number of vessels observed, being independent of image scale, and being easier to implement. Knudtson used the six largest arterioles and venules measured from photographs to calculate CRAE/CRVE in their paper, meanwhile we adopted four largest vessels (the largest vessel in each quadrant) to calculate CRAE/CRVE because the revised formulas were not affected by the number of measured vessels. The higher value of CRVE measured from the ONH cube scan was evidence of dilated retinal veins in HAPC patients. As we known, blood flow was adjusted by vessel diameter, even tiny change of vessel diameter would have a huge impact on blood flow. But vessel diameter alone is not sufficient to account for changes in blood flow. Therefore, CRAE and CRVE were invented to describe blood flow and caliber of vessel. Moreover, PaO_2_ plays an important role in adjusting of the vessel diameter and blood flow in human organs. Lower PaO_2_ in HAPC patients cause vasodilation effect in order to ensure normal blood supply in important organs. And the significantly dilated vessels further confirmed the well-recognized increased blood flow induced ODE in HA. CRAE was comparable between groups which suggested that chronic hypoxia has little effect on retinal artery caliber.On the other hand, as reported by Wang et al. [41], the arteriovenous difference in diameter may be helpful in delivering oxygen from the retinal circulation to retinal tissue in hypoxia situation.

Pearson correlation analyses disclosed that HGB concentration was positive correlated with CRVE, which could be understood as increased blood viscosity in HAPC patients should be responsible for dilated retinal veins. However, other parameters were independent of HGB. A prior study suggested the retinal circulation autoregulated in response to PaO_2_ and PaCO_2_ in HA [42], hence we should perform research to study the PaO_2_ in HAPC patients and to relate PaO_2_ with OCT parameters in the future.

Potential limitations of the current study should be considered. First, this cross-sectional study only included a limited number of participants. Second, there could be bias in the OCT parameters because some parameters were auto-calculated by built-in software which could display abnormal results. Third, We did not analyze the effect of HA living history, HAPC history, ethnicity and altitude on the retinal parameters. Forth, Age could be one of the confounding factors. As we know, RNFL thickness, ONH parameters and vessel diameters could be changed with age. Although we did not include age as a risk factor, we selected participants of age between 18 and 65 years to minimize the age bias. Another confounding factor could be refractive error. We excluded participants with spherical equivalent (SE) of exceeding ± 3.0 diopters to minimize the effect. Finally, Hospital of Maqing County has OCT equipment (ZEISS), hospital of Dawu County does not have OCT equipment so we brought our OCT equipment to local hospital to collect data. Therefore, there could be differences between two different OCT equipments.

## Conclusions

In conclusion, the retinal structure of HAPC patients had the following characteristics: thickened RNFL, enlarged ONH and dilated retinal veins. Moreover, increased blood viscosity caused by HGB should be responsible for dilated veins, but not for thickened RNFL and enlarged ONH. CRVE and CRAE calculated by revised formulas from ONH image of OCT scan was a reliable method to evaluated vessel and can be used in the future study. Our study may shed light on novel hypothesis for the pathophysiology of HA-related retinopathy.

## Data Availability

The datasets analyzed during the current study are available from the corresponding author on reasonable request.

## Abbreviations

HA: High altitude
HAPC: High altitude polycythemia
RNFL: Retinal nerve fiber layer
HGB: Hemoglobin
ONH: Optic nerve head
SD-OCT: Spectral domain optical coherence tomography
RVD: Retinal venous diameter
RAD: Retinal arterial diameter
HAR: High-altitude retinopathy
ICP: High intracranial pressure
ODE: Optic disc edema
AMS: Acute mountain sickness
CRAE: Central retinal artery equivalent
CRVE: Central retinal vein equivalent
AVR: Artery/vein ratio
